# A qualitative study on gender relations and digital payments: healthcare workers’ experiences during polio vaccination campaigns in Uganda and Malawi

**DOI:** 10.1136/bmjgh-2024-017475

**Published:** 2025-07-16

**Authors:** Paul Bukuluki, Simon Ndira, Juliet Aweko, Angelica Kiwummulo, Michael Ediau, Justine Namakula, Elizabeth Ekirapa-Kiracho, Peter Waiswa, John J O Mogaka, Grace Bantebya

**Affiliations:** 1 School of Social Sciences, Makerere University, Kampala, Kampala, Uganda; 2 Compelling Works, Kampala, Uganda; 3 Department of Health Policy, Planning and Management, Makerere University School of Public Health, Kampala, Uganda; 4 Department of Health Policy, Planning and Management, Makerere University Digital Payment Unit, Kampala, Uganda; 5 School of Public Health, Makerere University College of Health Sciences, Kampala, Uganda; 6 School of Public Health, Makerere University, Kampala, Uganda; 7 Women and Gender studies, Makerere University, Kampala, Kampala, Uganda

**Keywords:** Immunisation, Vaccines, Poliomyelitis, Qualitative study, Gender-Based Violence

## Abstract

**Background:**

Gender is a key factor shaping societal roles and access to resources, with cultural norms often limiting women’s abilities to use digital financial services. Despite the rise of digital payments, little is known about how gender relations influence their adoption and experience, especially among health workers involved in vaccination campaigns.

**Method:**

Between January and September 2023, we explored how gender norms and relations influence the uptake and experiences of digital payments among health workers participating in mass polio vaccination campaigns. The qualitative study involved 23 focused group discussions (FGDs; 16 in Uganda and 7 in Malawi) and 82 in-depth interviews (IDIs; 35 in Uganda and 47 in Malawi) with healthcare workers who received digital payments for implementing polio mass vaccination campaigns. IDI participants included village health teams, midwives, nurses, health facility managers, immunisation focal persons and district health officers in Uganda, and community health workers, health facility managers and mobile money operators in Malawi. FGDs were held with midwives, nurses and village health teams in Uganda and community health workers in Malawi. Data were coded using Dedoose software and thematically analysed.

**Results:**

Participants highlighted that digital payments were convenient since they were able to receive funds without travelling long distances or queuing at health facilities. Women reported that it gave them more time to engage in alternative activities, improve their financial autonomy and ability to participate in decision-making around use of household funds. Structural challenges leading to delayed disbursement of funds were reported to reinforce gender norms around financial dependency on men to meet operational campaign costs (eg, transport). Reported limited ownership of mobile money accounts, a prerequisite for digital payments, led to the exclusion of some women.

**Conclusion:**

Our findings suggest that digital payments could help improve financial autonomy and participation in decision-making around use of household funds among women involved in immunisation campaigns. However, our findings show that digital payments are implemented in the context of prevailing harmful gender norms that, if not addressed, have the potential to compromise women’s agency. This underscores the importance of integrating gender-transformative programming in planning for digital payments during vaccination campaigns.

**WHAT IS ALREADY KNOWN ON THIS TOPIC**

- Adopting digital payment systems during immunisation campaigns has the potential to improve the accessibility, efficiency and transparency of payments to healthcare workers in some settings.
- Cultural and gendered norms may constrain the roles women play in society, and power relations can influence their capacity to optimally use and benefit from digital financial services. However, there is limited evidence about how gender roles and social norms affect access to digital payment and use of funds obtained from digital payment during immunisation campaigns.

**WHAT THE STUDY ADDS**

- Our findings illustrate that while technologies like mobile money could potentially empower women by facilitating direct access to funds, structural barriers and cultural norms continue to dictate who has control over financial resources, reinforcing traditional power dynamics between gender groups.
- Our study also shows that the digital payment systems in polio vaccination campaigns do not give adequate consideration to gender dynamics and relations rooted in gender norms that have the potential to affect health workers’ control over earnings from digital payments and ultimately their motivation to participate in these campaigns.
- Lastly, our study demonstrates how women balance their expected gender roles and the demands of the work during the mass immunisation campaigns to ensure that they maximise the benefits of digital payment.

**HOW THIS STUDY MIGHT AFFECT RESEARCH, PRACTICE OR POLICY**

- The study emphasises the importance of gendersensitive planning in mass immunisation campaigns to ensure women benefit from digital payments, increase women’s participation and address potential gender-related challenges.

## Introduction

 Digital payments have been widely embraced in most health systems globally, and they have transformed payment systems and increased timely access to funds by the workforce in various sectors, including the health sector.^1^ They are perceived to be fast, convenient, traceable, reliable, easy and reasonable to set up.^1 2^ Subsequently, digital financial systems have been rolled out in some sub-Saharan African countries, including Côte d'Ivoire, Ghana, Kenya, Mali, the Democratic Republic of Congo and Nigeria.^3^ Existing evidence indicates that digital payments have also been used to improve the coverage and quality of vaccination campaigns.^1^ Digitised payment in the form of mobile money payment has been used increasingly in both Uganda and Malawi. In Uganda, approximately 43.3% people aged 10 years or older are registered on mobile money platforms.^4^ In Malawi, 60% of the population was registered on mobile phones.^5^ The Ministry of Health (MoH) in Uganda and Malawi, and some non-governmental organisations (NGOs) as well as bilateral and multilateral organisations in both countries, use mobile money to make payments to health workers attending workshops, training and those involved in large-scale health campaigns. In both Malawi and Uganda, the health workforce comprises trained formal health workers such as nurses and doctors as well as community health workers (CHWs), referred to as health surveillance assistants in Malawi. While CHWs are recognised as government employees who receive a salary in Malawi. In Uganda, they are still largely volunteers and are not yet salaried government employees. CHWs play a very active role in vaccination campaigns in both countries. Payments during these campaigns often comprise allowances which may be paid by cash or increasingly through digital payment. Anecdotal evidence suggests that during recent polio campaigns and the COVID-19 mass vaccination campaigns, digital payments were heavily used in both countries to pay campaign health workers. Officials in the MoH and the WHO country office identified several factors that constrained digital payment use. They included (1) unnecessary administrative burden resulting from the need to generate a list of beneficiaries to be paid for every event, (2) delays in making payment to beneficiaries as the validation of their mobile money numbers takes a while and (3) payments to the wrong persons due to errors in the validation process.

A recent study on the adoptability of digital payments for CHWs in peri-urban Uganda revealed that determinants of digital payment uptake included perceived risk of the payment method and trust in the system.^6^ Furthermore, another recent study in Uganda investigated if cash or digital payment modalities affect CHW performance. The study showed the factors which influenced and hindered performance. However, overall, it found that CHWs preferred and performed better with cash payments because digital payments were associated with delays and payment shortfalls that demotivated them.^7^ However, another previous study noted that payment digitisation efforts in the health sector in low/middle-income countries have accelerated due to the COVID-19 pandemic, and it (wage digitalisation) could potentially improve health system performance and provider well-being and consequently, patient outcomes. It further notes that critical gaps in evidence need to be addressed to support implementation and effective innovation.^8^

Nevertheless, few studies have paid attention to the intersection of digital payments and gender relations and norms and how gender relations affect the adoption and use of digital payments in relation to health worker motivation and performance that is essential for increasing coverage and quality of vaccination campaigns. According to Cislaghi and Heise, ‘gender norms are social norms defining acceptable and appropriate actions for women and men in each group or society’.^9^ They are embedded in formal and informal institutions, nested in the mind and produced and reproduced through social interaction. They play a role in shaping women and men’s (often unequal) access to resources and freedoms, thus affecting their voice, power and sense of self.^9^ However, social norms are defined as ‘rules of action shared by people in a given society or group; they define what is considered normal and acceptable behaviour for the members of that group’.^10^

Therefore, given the centrality of gender norms in regulating relationships between men and women and their behaviours in various contexts, existing gender norms could potentially influence the extent to which digital payments contribute to increased motivation and performance of health workers.^11^ Additionally, they could also shift gender relations. Women CHWs play a critical role in linking communities to key health services and delivering basic, often life-changing health information and education, particularly in settings where societal gender norms constrain or prohibit communication between women and men.^12^ A significant number of the CHWs are women, and yet evidence from most resource-constrained economies suggests that women have poorer access to and use of digital financial services (DFS) than men.^2^ Contributing factors include limited phone ownership and access to the internet, inadequate access to financial resources and financial and digital illiteracy.^13 14^ The existence of gendered social norms, attitudes and legislation overwhelmingly underpin factors that prevent women from accessing DFS.^15^ Gender is a central organising factor in societies, and it can significantly affect the processes of production, consumption and distribution.^16^ Cultural norms frequently constrain women to specific societal roles, and gendered norms influence women’s capacity to use digital financial services.^11^ Uganda is generally considered a patriarchal society, where men typically dominate household decision-making and control resources, often serving as the primary breadwinners.^11^ Women are traditionally expected to be submissive to their husbands and primarily responsible for child-rearing and household chores such as cooking and cleaning. However, these gender roles are gradually evolving, particularly among more educated individuals and those involved in politics.^17^ In contrast, Malawi presents a more complex picture, with both matrilineal and patrilineal communities. While women in matrilineal groups can inherit property, overall decision-making remains predominantly controlled by men across the country.^17^

The gender norms described above in Uganda are therefore also prevalent in most of Malawi.^10 18 19^ Both countries have legal instruments for promoting gender equality, although their implementation has remained weak.^20 21^ The participation of women in economic activities is mainly within the informal sector and agricultural sector in both countries. This study was conducted during polio vaccination campaigns in Uganda and Malawi, when both countries faced polio epidemics and were participating in a WHO-led digital health payment programme. This study aimed to explore the influence of gender norms and relations on digital payment uptake and experiences of health workers involved in the mass polio vaccination campaigns within these specific settings.

## Methods

This paper is based on synthesis of data from qualitative studies conducted in Uganda and Malawi following the 2022 polio campaigns in the two countries. The studies aimed to understand the influence of gender norms and relations on digital payment uptake and experiences of healthcare workers involved at different levels in the recent polio immunisation campaigns in the two countries.

### Study conceptual framework

The study applied the Morgan *et al* gender analysis framework^22^ shown in table 1 to thematically analyse and present the data from the two countries. The framework argues that sex disaggregation alone is not sufficient in showing differences and similarities among men and women in health systems research. Further analysis and questions to understand how gender power relations are constituted and negotiated are paramount. Understanding gender power relations is hinged on four critical domains that include; (1) examining who has what (access to resources); (2) who does what (the division of labour and everyday practices); (3) how values are defined (social norms); and (4) who decides (rules and decision-making) and these are influenced by individuals and their environment.

**Table 1:** Framework for gender analysis by Morgan *et al*, 2016

What constitutes gendered power relations	
Who has what	Access to resources (education, information, skills, income, employment, services, benefits, time, space, social capital, etc)
Who does what	Division of labour within and beyond the household and everyday practices
How are values defined	Social norms, ideologies, beliefs and perceptions
Who decides	Rules and decision-making (both formal and informal)
How power is negotiated and changed individual/people	Critical consciousness, acknowledgement/lack of acknowledgement, agency/apathy, interests, historical and lived experiences, resistance or violence
Structural/environment	Legal and policy status, institutionalisation within planning and programmes, funding, accountability mechanisms

**Figure 1:**
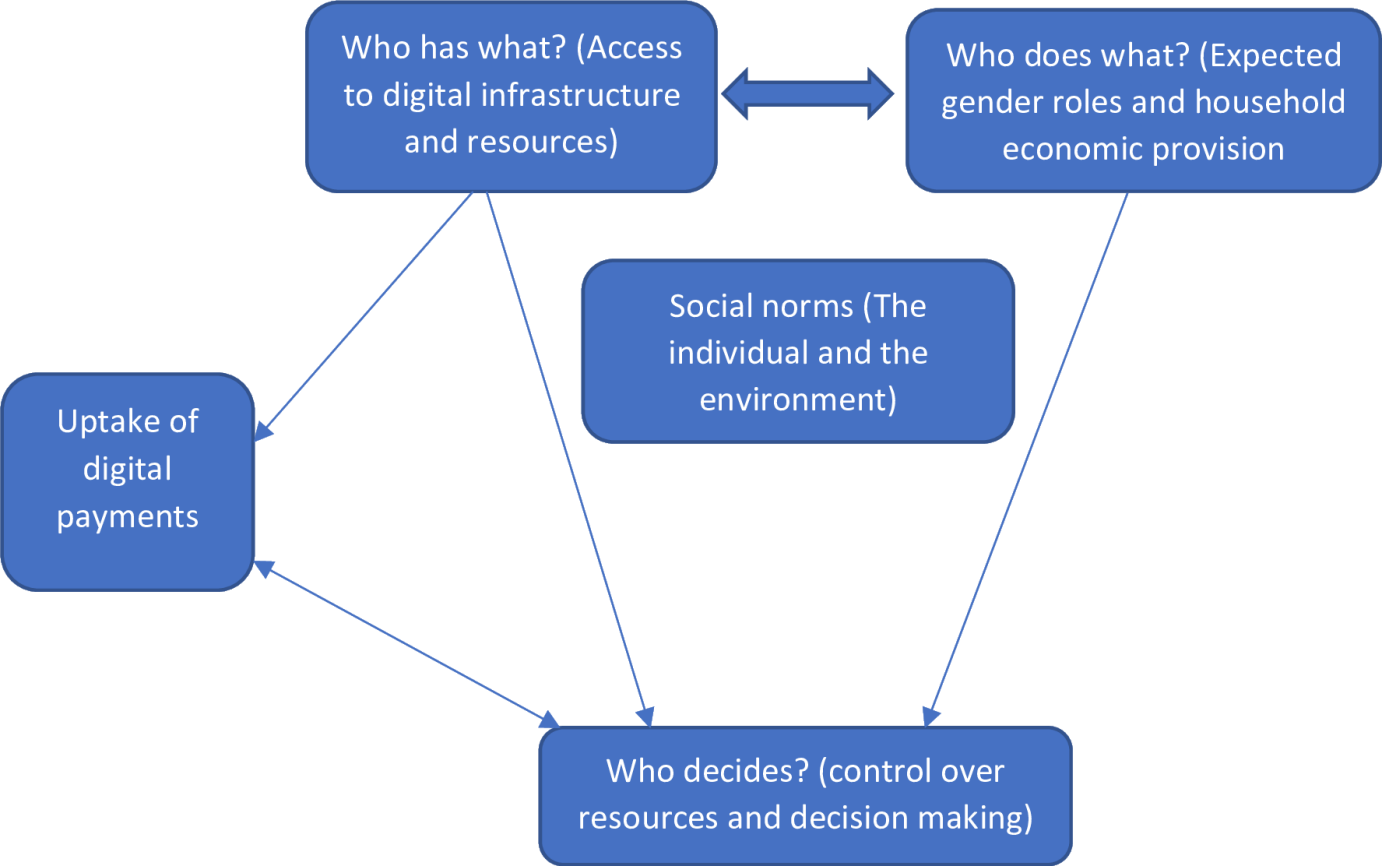
Interpretation of gender power relations and uptake of digital payments among vaccination health workers in Uganda (adapted from Morgan *et al* framework).

Figure 1 provides a visual summary of how we applied this framework. Access to digital payment resources including phones and registered mobile money accounts determines the digital resources that men and women have access to (who has what). Ownership of these resources influences what men and women are able to do by influencing and reshaping existing gender norms and values (who does what). However, the latter (who does what) also influences the former (who has what). For example, if women can also work and earn mobile money then they can buy and own mobile phones. Similarly, control over resources (who decides) is influenced by both who has access to these resources and what they are able to do now that they have these resources. Lastly, the uptake of digital payment services is affected by both who has access to specific digital resources and who controls the use of resources such as phones and mobile money accounts.

### Study setting

The study was conducted in four randomly selected districts, from three regions in Uganda: the Northern region (Lira district and Kitgum district), Central region (Rakai district) and Eastern region (Iganga district). In Malawi, the surveyed districts are located in three regions of Malawi: the Northern region (Mzimba and Mzuzu City), the Central region (Nkhotakota and Ntcheu) and the Southern region (Mangochi, Mwanza and Zomba City). In both countries, agriculture is predominantly the main economic activity in the study districts, with the majority of households engaged in the subsistence economy.^4^ About 4 out of every 10 persons own (43.3%) a telephone in Uganda.^4^ In Malawi, approximately 60% of the population had a mobile telephone.^5^

### Study participants and selection criteria

In both countries (Uganda and Malawi), study participants were purposively selected and comprised health workers who implemented the recent polio mass vaccination campaigns and also received payment for their service through mobile money (digital) or a cash-based system more than once. In Uganda, participants (health workers) included village health teams (VHTs), midwives, nurses, in-charges (heads) of health facilities, polio immunisation focal persons and district health officers/assistant district health officers, midwives, nurses and VHTs. In Malawi, participants specifically comprised the CHWs who participated in the polio mass vaccination campaign in the selected health centre catchment areas of each of the seven study districts. Additionally, we included health system managers and mobile money company representatives at district and central levels.

The study districts were selected to represent peri-urban, urban and rural settings. Similarly, the health facilities in the research districts were selected using the maximum variation (maximum heterogeneity) approach, including rural and urban settings and taking into consideration socioeconomic factors. All participants were purposively selected based on their experience in the polio vaccination campaign or the mobile money payment system. We also purposively selected both men and women polio vaccination campaign health workers from the respective districts to enable investigation of the role of gender norms in influencing the response of health workers to digital payments in the polio immunisation campaigns. Purposive sampling provides an opportunity to select a group of people that match the characteristics relevant to the research question and that have experiences that are relevant to the study. However, purposive sampling does not permit findings to be generalised to the entire population.^23^

### Patient and public involvement

Neither patients nor the public were involved in the design, or conduct, or reporting or dissemination plans of this research.

### Data collection methods and tools

The study used qualitative data collection methods in both Malawi and Uganda study sites. Focused group discussions (FGDs) were used to obtain the different perspectives of digital payments, gender norms and relations among the participants while the in-depth interviews (IDIs) enabled us to explore detailed personal feelings and experiences about the subject matter.

We conducted 23 FGDs (16 in Uganda and 7 in Malawi) with midwives, nurses, CHWs and VHTs in Uganda and Malawi, respectively, and 82 participants participated in the IDIs (35 in Uganda and 47 in Malawi).

Separate semi-structured interview guides were used for the FGDs and IDIs. The guides were tailored to the diverse categories of participants and included questions that aimed to elicit key themes, including gender norms, gender relations, health workers’ experiences of digital payments motivation and drawbacks, perceived effect of digital payments on gender roles, gender norms and health workers’ response (motivation) to digital payments.

Research assistants (RAs) who had experience in conducting qualitative interviews and FGDs were recruited and trained to ensure they were able to collect quality data. For IDIs, the RAs conducted one-on-one (face-to-face) interviews with participants. Two RAs conducted each FGD. They included the lead interviewer and note-taker who was responsible for taking notes. Each FGD consisted of 6–12 participants and had men and women combined. All IDIs and FGDs were audio-recorded. The IDIs lasted approximately 1 hour (60 min) while FGDs lasted 1½ hours (60–90 min). All interviews were largely conducted in English, except for those with some CHWs in Malawi, which were held in the local language. IDIs and FGDs were conducted until information saturation was reached: no new information and unique experiences were emerging from the study participants.

### Data analysis

All data in the form of audio recordings from IDIs and FGDs were transcribed verbatim, translated into English (interviews with CHWs in Malawi) and transcripts were entered into the Dedoose qualitative data management and analysis software. The data was coded in the respective countries. Through an iterative process involving debriefing and member checks, the codes were reviewed, interpreted and categorised according to three main domains of Morgan *et al*’s gender analysis framework^22^ (who has what?, who does what? and who decides?) as depicted in figure 1.

## Results

The data yielded three main themes and two subthemes describing the influence of gender power relations on digital payment uptake among polio vaccination campaign health workers in Uganda and Malawi.

### Access to digital infrastructure and resources (who has what?)

According to the prevailing social norms, men are often perceived to have more ease in accessing digital tools, especially phones that aid digital payment, compared to women. This was demonstrated through two main themes: Gender variation in ownership of phones and mobile money accounts and Dependency on men for financial resources.

#### Gender variation regarding the ownership of phones and mobile money accounts

Data from the study revealed significant disparities in beliefs about mobile phone ownership and access to digital resources among health workers in Uganda and Malawi. The findings indicated that whereas no one questioned whether men should own phones, some men in Uganda were not comfortable about women owning phones, and yet this affects who actively engages with and uses technology. In Uganda, some men who participated expressed scepticism regarding women owning phones, viewing such ownership as problematic and associated with mistrust and loss of control over women since some men could not determine how the women used the phones and for what purpose.

A woman who owns a phone is very disrespectful. Man VHT, Lira, Uganda

This statement reflects the belief that men should control such resources, leading to barriers that restrict women from accessing technology. Some women reported that they did not have personal phones because their husbands prohibited them from owning one.

Some women do not have phones because their husbands do not allow them to have phones… IDI Man District Health Management Team (DHMT, Lira, Uganda.

Some key informants highlighted that the requirements for registration for mobile money accounts were cumbersome for some health workers, particularly women, as they lacked the necessary documentation including national identification cards.

Some women do not have national identification cards, making it difficult for them to register for mobile money, forcing them to use other people’s phone numbers." Man DHMT, Lira, Uganda

#### Dependency on men for financial resources

While some participants noted that mobile money systems provide women with relatively easier access to financial services, they also highlighted instances of dependency and control. In Uganda, women health workers often registered their mobile money accounts using their husbands’ numbers to overcome these barriers. However, this often resulted in limited access to their own funds and limited control over financial resources.

I had registered with my husband’s number, so he is the one to receive the money. By the time he tells me about it, he has already used the money… FGD Woman Health Worker, Rakai, Uganda

Similarly in Malawi, women CHWs highlighted that their reliance on their spouses’ phones resulted in limited access to funds.

I used my husband’s phone number. When the allowances were deposited, my husband withdrew the money and used it without letting me know. When I asked him about this he became furious and this has led to conflicts in our households. FGD-CHW Lilongwe, Malawi

Challenges in programming that led to delayed digital payments also reinforced the extent to which women depend on men for financial resources. Women health workers reported that delays in receiving payments or failure to receive payment before the campaign for costs such as transport forced them to seek assistance from their spouses or friends, further entrenching their reliance on men’s financial support.

This illustrates that while technologies like mobile money could potentially empower women by facilitating direct access to funds, structural barriers and cultural norms continue to dictate who has control over financial resources, reinforcing traditional power dynamics between genders.

### Expected gender roles and economic provision in households (who does what?)

In both Malawi and Uganda, traditional social norms dictate that men are primarily responsible for financially providing for the household, while women are expected to manage domestic duties. Several participants indicated that the digital payment system has enabled women CHWs to balance their professional responsibilities with household chores. Digital payments enable them to avoid frequent trips to district offices to collect cash, avoiding unnecessary travel away from home and its associated costs. As a result, they can focus on completing their household chores or other related activities in their homes while waiting for payment notifications, minimising disruption to their schedules. Participants noted that this shift saves time, allowing them to engage more effectively in both professional and domestic tasks.

The digital payment system helps in saving our time. Sometimes we receive our allowances while we are at home, which gives us enough time to do our household chores and other tasks before going to a mobile money agent. Unlike the cash payment period, when we had to excuse ourselves from chores to travel to receive allowances. FGD-CHW-Mzuzu-45867-46319, Malawi

This change is set against the backdrop of prevailing cultural attitudes that historically positioned men as the primary earners. The shift in gender norms has increased the expectation for women to contribute to the financial sustainability of the household. Campaigns involving health workers included both men and women, and both groups were expected to earn an income through digital payments thereby increasing household income and enhancing relational dynamics within the household.

When a woman is earning, there is peace in that home/family. She gets the opportunity to support the family better. Man DHMT member, Lira, Uganda.

While some adjustments to these norms have emerged to allow women’s participation, there are still underlying tensions. For instance, findings from both Uganda and Malawi showed that while some men support participation of their spouses in vaccination activities, there are fears that women’s earnings would take on some of the financial responsibilities of men shifting the burden to women. Some women reported that their husbands’ recognition of their income led to a shift in responsibilities, with men reducing their own economic activities under the assumption that the woman’s income would suffice, thus increasing pressure on the women.

It has put me at a disadvantage in my household. My husband stopped engaging in any income-generating activities once he learned I was receiving allowances. We ended up relying on the allowance to buy all household necessities, which is not sufficient and places me under pressure.” FGD-CHW-Rumphi, Malawi.

Interestingly, some women CHWs reported that having their mobile money accounts provided them with increased privacy regarding their personal finances, allowing them to manage their income without their husbands’' knowledge.

Since I am paid via mobile money, my husband might not know that I have received the money. I can even delete the transaction messages. If I were paid in cash, my husband could easily collect it and misuse it.” FGD-HH-Mzimba-27708-2824, Malawi.

The convenience and speed offered by digital payments have been perceived as helpful in alleviating mistrust that arises from the existing social norms that do not expect women to stay away from their homes for long periods if they are not engaging in clearly defined tasks. Some women CHWs had reported that in the past when they stayed away from their homes for long periods, awaiting their cash payments, their husbands had falsely accused them of infidelity.

When we received money by hand and spent the whole day at the collection point, our husbands would often think we were out having an affair. The mobile money payment method has simplified this, as we receive notifications directly. FGD-CHW-Chikwawa, Malawi.

Failures or delays in the payment system, if not communicated early and clearly can lead to negative repercussions for couple relationships. Men, who anticipated immediate receipt of funds on completion of vaccination campaigns, in some cases suspected their partners of wrongdoing when payments were delayed. The respondents reported that such scepticism could lead to diminished appreciation of the economic contributions and may instead cause the men to deny their wives permission to participate in future campaigns.

Mobile money payments can cause bad relations between partners due to delays, as the male partner may think that the woman has already received the payment and misused the money. Man VHT, Kitgum, Uganda.

This causes gender-based violence; a man may not permit a woman to go to work if there is no payment, deeming the work useless. Woman VHT, FGD Kitgum, Uganda.

### Decision-making and resource control at household (who decides?)

In both Malawi and Uganda, prevailing social norms dictate that men are the primary decision-makers and controllers of economic resources within households. Women are often expected to seek permission from their husbands for financial decisions, which reinforces a patriarchal structure that prioritises men’s authority. Despite this backdrop, some participants felt that digital payment systems have played a role in empowering women to take a more active role in financial decision-making at the household level since it has given them an opportunity to contribute to decisions about how the money is spent at household level.

For us household members, I can say that mobile payment has made it possible for us to take part in decision-making because we know how much has been received. Therefore, we are involved in budgeting the money. FGD4-HH-MAERA-ZOMBA-15570-15948, Malawi.

We make decisions as husband and wife. I present the money and explain that this is the money from the immunization work we did; therefore, we prioritize whether it should go for school fees, foodstuff, or gardening, depending on the relationship at the time when I receive this money. FGD woman VHTs, Kitgum district, Uganda.

However, the study also revealed tendencies of unequal control over e-money, as ingrained gender norms continue to emphasise male privilege in decision-making and resource control. Many participants highlighted the pervasive risks of intimate partner violence, especially in situations where women did not notify their husbands about receipt of payments or disagreed with husbands on expenditure decisions. Intriguingly, men were generally not expected to inform their wives about any money they received or to consult them on its use.

It is the man who makes the decision. So, after getting the money, it is the man who decides how the money shall be spent. Woman Health worker, Iganga, Uganda.

Some homes where a woman freely spends her money without notifying the man, and the man also does the same, this is causing a lot of gender-based violence. Man DHMT, Lira, Uganda.

On the other hand, the study found that digital payment platforms have afforded women a greater sense of autonomy in decision-making regarding the use and allocation of funds. Multiple participants argued that mobile money payments empower them to control and protect their financial transactions, as they can choose not to disclose their Personal Identification Number or password. Furthermore, women often own their phones, making it difficult for spouses who are men to access funds without the woman’s consent.

I have control over my money because it’s me who receives the message, then I am the one to decide whether to tell the man that I have received it or not. Woman health worker, Rakai, Uganda.

However, this autonomy can come at a cost. Women health workers’ non-disclosure of the funds they receive can increase the risk of intimate partner violence. Husbands may accuse their wives of lacking transparency in financial matters, suspecting them of infidelity.

[…] So he will hear from someone that the money has already been paid, yet you have already used the money […] and asks you, have you received the money… and that causes problems. Woman Health Worker, Kitgum, Uganda.

Interestingly, many participants noted that women who disclose their digital payments to their husbands often enjoy better relationships. Open communication creates opportunities for renegotiating power dynamics around fund usage, fostering more collaborative decision-making.

What brings tension in families is when a woman doesn’t want to open up (about mobile money payments) to a man, but if she is free and shares freely, usually the relationships are good… Man VHT, IDI Lira, Uganda.

When money is sent on mobile money, I will still take the responsibility to go withdraw and bring it home so that we agree on how to spend the money. Woman VHT, Lira, Uganda.

Trust and communication among couples prove vital in determining whether women disclose their financial transactions from digital payments to their spouses. Participants observed that relationships characterised by peace, trust and understanding create a conducive environment for open financial discussions, while tumultuous situations inhibit sharing.

[…] She might not inform her husband when there is no peace at home, but when there is peace, she will openly tell him that she has received the money. Woman Health Worker, Iganga, Uganda.

It depends on one’s home. Some men can hide certain things from their women, including mobile money payments. Man DHMT member, Lira, Uganda.

Ultimately, findings demonstrate that digital payments have the potential to enhance women’s voices within household spending decisions. Digital payment systems often include tools for tracking expenses and budgeting, which assist families in managing their finances more efficiently. Women have leveraged these tools to contribute significantly to household spending and saving decisions.

We have seen a change with the presence of mobile payment because now we can budget on how we are going to use the money while it is still in our phone. This has enabled us to budget accordingly. FGD-HH-KUNENEKUDE-Mwanza-6440-6605, Malawi.

The benefit of mobile payment I have seen in my household is that we can budget appropriately before even withdrawing the money. If we need to use some of the money, we know the exact amount of money we want to use and we withdraw that. FGD-HH-MAERA-ZOMBA-6901-7139, Malawi.

## Discussion

Our findings highlight five key points that underline the complex interplay between gender power dynamics, technology and financial empowerment in the context of digital payments among health workers in Uganda and Malawi.

### Gender disparities in digital ownership and access

Gender disparities in digital ownership and access present significant obstacles for women in sub-Saharan Africa. Studies demonstrate that sociocultural norms often favour men, limiting women’s engagement with digital technology. Previous work has noted that societal expectations frequently prioritise men’s control over resources, severely impacting women’s access to mobile phones and financial services.^24^ Our study reinforces this, revealing that participants in Uganda and Malawi perceive digital payments as more beneficial than traditional cash-based payments, as they provide more advantages to women with regard to direct access and control over their income. For women who are health workers, specifically, digital payments facilitate a greater ability to negotiate and make decisions on spending, as they receive funds in password-protected mobile money accounts that limit or exclude access by their spouses. This growing autonomy is vital in shifting gender norms concerning access and control of financial resources and enhances women health workers’ motivation to participate in vaccination campaigns. This finding is in agreement with existing evidence that digital financial services can enhance women’s social and financial autonomy.^25 26^

This shift in resource control can lead to a more collaborative approach to financial decision-making among partners. Digital payments create a transparent record, making women’s economic contributions to the household more visible and acknowledged. This recognition may support more balanced gender dynamics regarding ownership and access to digital resources.

### Dependency on men for financial resources

Despite the advancements brought about by digital payments, women’s reliance on men remains a pervasive barrier to their economic independence. This trend, documented extensively in other studies, shows that many women depend on relatives, mainly men, for financial access, thereby limiting their ability to make independent choices.^27^ This dependency shifts the power dynamics, positioning men as gatekeepers to financial resources. In our study, women health workers often felt obligated to involve their men counterparts in financial matters, reflecting the existing authority that men maintain within domestic settings. While the digital banking environment may enhance women’s financial autonomy, entrenched gender norms continue to pose challenges. A significant finding is that when income from digital payments is disclosed to spouses, the latter often maintain significant influence over how these funds are used, thereby reinforcing patriarchal norms. Moreover, non-disclosure of earnings can lead to increased risks of intimate partner violence (IPV).^28^ This complex interaction reveals a critical dilemma: while digital payments can enhance autonomy, they also require careful navigation of existing gender-related pressures, which may discourage participation in health campaigns.

### Impact of digital payments on gender roles

Digital payment systems hold great promise for enhancing women’s economic participation. The World Bank^29^ reports that mobile money services empower women by enabling them to engage more actively in economic activities. From our findings in Uganda and Malawi, digital payments have demonstrated potential for shifting gender norms related to men being seen as the sole breadwinner. The contribution of women to meeting household costs was acknowledged and appreciated by some of the families. Our findings also illustrate that digital payments increased opportunities for women health workers to make household decisions regarding household earnings, as evidenced by the increased opportunities for shared decision-making. Such dynamics introduce avenues for negotiation and collective decision-making, highlighting the transformational potential of digital payments. However, our research also points out important limitations. Increased economic participation through digital payments can place additional financial responsibilities on women, often leading to stress and exacerbating existing burdens.^30^ This is more so if the men are laid back and do not take additional responsibilities at home to assist with domestic chores or take on additional income-generating activities.

We noted that the scheduling of immunisation activities is frequently insensitive to gender norms, limiting women’s ability to participate fully. Many women reported needing permission from their spouses before engaging in these activities, especially when notifications about vaccination campaigns are received on short notice. Women reportedly felt pressured to fulfil their gendered household tasks and also accomplish the vaccination activities. This was often more difficult if they had to provide vaccination services very far from their homes, because it meant they had to wake up very early to first complete tasks at home. Current arrangements for digital payments often lack the foresight required to ensure safe and equitable participation of women, potentially leading to tensions and conflicts that undermine women’s autonomy and opportunity to participate in gainful work.

### Communication and trust in financial disclosure

Effective communication about financial matters is essential for fostering healthy relationships. Our findings corroborate prior work emphasising how open discussions between spouses can enhance trust and satisfaction.^31^ However, lack of transparent communication regarding financial matters can contribute to power imbalances and, in some instances, may increase the risk of IPV when women are unable to disclose or discuss their earnings freely. On the other hand, our work revealed that spouses who had healthy relationships and open communication were able to avoid conflict and tended to disclose and plan for their income peacefully. The ambivalence surrounding financial matters and social norms related to disclosure necessitates frameworks that encourage open financial dialogues between partners to promote fairness in decision-making.

### Implications for organisers of campaigns and implementers of digital payments

The underlying social and gender norms regulating relationships between men and women tend to influence how men and women who are health workers benefit from digital payments and, ultimately, their motivation and potential to contribute to such mass campaigns positively. Current arrangements for digital payments are largely gender-blind and do little or nothing to prepare health workers to deal with the backlash from gender norms likely to be experienced when funds are received through digital payment modalities. Therefore, our data imply that planners and implementers of immunisation campaigns and digital payments should ensure they are cognisant of such power imbalances rooted in the gender norms. Second they need to provide timely communication about any changes regarding payment to minimise gender-related conflicts regarding payment. Without integrating gender-transformative activities in digital payments, there is potential to compromise women’s agency by reinforcing harmful gender norms that influence women’s roles and responsibilities. Through digital payments, women save time which is likely to be used to further engage in domestic chores and other activities that empower them with new knowledge, skills and engagement in alternative livelihood activities. Although our results report that, some men take a step back and reduce their economic activity when their spouses earn income through digital payments without necessarily taking on additional household responsibilities, it is imperative to integrate gender transformative activities that engage men to ensure that the burden of household work does not shift to women but is instead shared between men and women.

It is also worthy to note that social and gender norms combine with other structural factors such as technological infrastructure and socioeconomic factors to impact the effective participation of women in fully engaging with digital payment systems. Working on social norms is not enough. Implementers engaging in digital payments require a focus on norms shifting as well as addressing key structural and institutional factors that limit women’s access to digital infrastructure. We acknowledge that our work did not explore the perspectives of spouses of community workers involved in vaccination campaigns. This limitation prevented us from capturing a deeper understanding of the household gender dynamics relating to digital payments. Additionally, we did not include the views of relevant stakeholders such as the Ministry of Gender and Social Welfare and other related programmes outside health which would have enriched the perspectives presented in our findings.

## Conclusion

Overall, our study demonstrates that while digital payments can enhance control for women health workers, entrenched gender norms and sociocultural pressures remain significant barriers that inhibit full participation and empowerment.

Addressing these issues requires collaborative efforts among governments, NGOs and community leaders to create supportive frameworks for women’s financial empowerment within health contexts. By challenging sociocultural barriers and systemic inequalities associated with digital payment systems, we can cultivate an environment that allows women to thrive formally and informally. Furthermore, planners and implementers of vaccination campaigns must ensure that gender dynamics are recognised and integrated into the design and scheduling of campaign programmes and execution of financial systems, allowing for the comfortable participation of women and long-term sustainability and effectiveness in health interventions.

## Data Availability

Data are available upon reasonable request.
